# Prevalence and predictors of self care practices among hypertensive patients at Jimma University Specialized Hospital, Southwest Ethiopia: cross-sectional study

**DOI:** 10.1186/s13104-019-4125-3

**Published:** 2019-02-14

**Authors:** Busha Gamachu Labata, Muktar Beshir Ahmed, Ginenus Fekadu Mekonen, Fekede Bekele Daba

**Affiliations:** 1 Pharmacy Department, Wollega University, Nekemte, Ethiopia; 2 Department of Epidemiology, Jimma University, Jimma, Ethiopia; 3 Pharmacy Department, Jimma University, Jimma, Ethiopia

**Keywords:** Hypertension, Predictors, Self-care practices

## Abstract

**Objective:**

Hypertension is a major risk factor and precursor of myocardial infarction, chronic kidney disease, heart failure and premature death. These vascular events increased costs of hypertension management. Self-care Practices were recommended to control blood pressure among hypertensive patients. Therefore, the objective of this study is to assess predictors of self-care practices among hypertensive patients at Jimma University Specialized Hospital.

**Results:**

A 341-hypertensive patients participated in the study. The mean age of the participants was 54.35 ± 12.48 years with range of 26 to 89 years. One hundred seventy-seven (51.9%) respondents were males and male to female ratio is 1.08. About 61.9% of respondents were adherent to medication usage and 30.5%, 44.9%, 88.3%, 93.5% and 56.9% of respondents were adherent to low salt diet, physical activity, non-alcohol drinking, nonsmoking and weight management respectively. Normal weight (AOR = 1.822, 95% CI 1.073–3.093) was independent predictor of medication usage whereas good self-efficacy (AOR = 2.584, 95% CI 1.477–4.521) and being female (AOR = 0.517, 95% CI 0.301–0.887) were independent predictor of low salt diet and physical activity respectively. Also being female (AOR = 3.626, 95% CI 1.211–10.851) was independent predictors of non-smoking.

## Introduction

Hypertension is a condition in which the blood vessels have persistently raised pressure and the average of two or more properly measured, seated blood pressure (BP) readings on each of two or more clinic visits is used [1]. Hypertension is a major risk factor and precursor of myocardial infarction, chronic kidney disease, heart failure and premature death. These vascular events increased costs of hypertension management [2].

About one-third of adults in the world have hypertension [3]. These are predicted to 1.56 billion by the year 2025 [4]. Ethiopian epidemiology of hypertension was not well studied. Nevertheless, in southwest Ethiopia, the overall prevalence of hypertension is 13.2% [5] while in Gondar city is 28.3% [6].

Self-care practices (SCPs) includes that the medication taking, non-smoking, weight management, low-sodium and low-fat diet, physical activity and moderate alcohol consumption [7]. Self-care is multidimensional as it relates to chronic disease management [8]. Adherences to SCPs were the similarity between recommended practice and actual practice [9].

Smoking cessation has immediate as well as long-term benefits for patients with hypertension, prevents cardiovascular disease and premature deaths [10, 11]. Similarly, reducing of dietary sodium intake less than 2400 mg/day and implementing dietary approaches to stop hypertension (DASH) through proper diet program like fruits and vegetables leads to reduce BP [10]. The literature studies revels that DASH diet reduced systolic BP by 8–14 mmHg, moderation of alcohol reduce systolic BP by 2–4 mmHg [12] and reduction in weight by 5–10 kg shows significant impact on systolic and diastolic BP [13]. WHO recommend at least 150 min of moderate-intensity aerobic physical activity throughout the week to lower BP [14, 15].

Patients who involved in SCPs benefit from the BP control, but adopting and maintaining SCPs for chronic disease management often require life-long practices, motivation and support [16]. Older age, female, self-efficacy and longer duration of hypertension were predictors of SCPs [16, 17]. Therefore, the objective of this study was to assess Predictors of SCPs among hypertensive patients on follow up at Jimma University Specialized Hospital (JUSH) ambulatory unit using adapted Hypertension Self-Care Activity Level Effects (H-SCALE) questionnaire [17].

## Main text

### Patients and methods

#### Study design and period

Hospital based cross-sectional study was conducted from April 4 to May 30, 2016.

#### Study population

Adult hypertensive patients on follow up in the ambulatory care unit of JUSH, and who were placed on treatment for more than 6 months were included in the study [18]. Patients unable to communicate and mentally ill were excluded from the study.

#### Sample size and sampling technique

Sample size was calculated using a single population proportion formula considering a 95% confidence level, margin of error (0.05), proportion of adherence with antihypertensive medication (P = 0.557) [19].

$${\text{n }} = \frac{{\left( {{\text{Z}}_{\alpha / 2} } \right)^{ 2} {\text{p }}\left( { 1- {\text{p}}} \right)}}{{{\text{d}}^{ 2} }}$$

The formula yields 380 hypertensive patients. Since the estimated total population of hypertensive patients was, less than 10,000 we used correction formula.

$${\text{nf}} = {{\text{n}} \mathord{\left/ {\vphantom {{\text{n}} {\left( {1 + \frac{{\text{n}}}{{\text{N}}}} \right)}}} \right. \kern-0pt} {\left( {1 + \frac{{\text{n}}}{{\text{N}}}} \right)}}$$

N = total targeted population on chronic follow up (2015).

Then the final sample size according to these equation yields 320 and adding 10% for nonresponse it becomes 352. Therefore, using patients’ card number 352 patients were recruited by simple random sampling technique from 2015 hypertensive patients and were interviewed after they re-fill their medication.

#### Data collection instrument

Sociodemographic, hypertension knowledge, and social support of patients’ data were obtained by structured questionnaire. Hypertension self-care practices were assessed by adapted H-SCALE questionnaire [17].

#### Ethical considerations

Approval for this study was obtained from the Institutional Review Board of Jimma University and JUSH clinical director in 2016. Written approval consent was obtained from literate participants and oral approval was considered in case of illiterate participants.

#### Operational definitions

*Self*-*care practice*: Is a framework for patient centred hypertension self-management and care.

*Self*-*efficacy*: A confidence in one‘s ability to participate in a given activities.

*Medication adherence*: Three items assessed the number of days in the last week that an individual takes medication, at recommended dosage and at same time. Responses were summed (range 0–21). Score = 21 were considered adherent.

*Low*-*salt diet*: six items assessed practices related to eating a healthy diet. A mean score is calculated. Scores of 6 or better were considered adherent.

*Physical activity*: Past 7 days physical activity of patients’ was assessed by 2 items. Responses were summed (range 0–14). Participants who scored ≥ 8 were adhering to physical activity.

*Non*-*smoker*: Respondents who reported 0 day smoking in the past 7 days.

*Alcohol*: Alcohol intake is assessed using 3-items. Participants who usually did not drink at all were considered abstainers.

*Weight management*: Seven items, strongly disagree (1) to strongly agree (5), assessed weight management. Responses were summed creating a range of scores from 7 to 35. Score ≥ 28 were considered adherent to weight management practices.

*Social support*: It was assessed with 12 questions and answers range from 12 to 60.

Range of 12–42 has low, 43–52 has medium and 53–60 has greater social support.

*Knowledge*: Assessed by 15 questions by giving 1 to correct answer and 0 to the wrong answer. Scores < 8 were taken as poor, 8–12 average, and 13–15 adequate knowledge of hypertension.

*Urban residence*: Patients who had town identification card.

## Results

### Characteristic of hypertensive patients

A total of 352 individuals were invited to participate in the study; out of them only 341 (96.88%) were fully responded. The mean age of the participants was 54.35 ± 12.48 years with range of 26 to 89 years. One hundred seventy-seven (51.9%) respondents were males. One hundred eighty-six (54.5%) were Muslim by religion and Oromo account 200 (58.7%). One hundred forty-nine (43.7%) were Illiterate. Married respondents account 279 (81.8%) and 182 (53.4%) live in Urban. One hundred twenty-two of respondents had estimated monthly income of 501–1500 Ethiopian birr (ETB). About 52% of respondents had medium social support. Two hundred thirty-seven (69.5%) of the participants were diagnosed to have hypertension before 3 years. Fifty-five (16.1%) of patients had diabetes as comorbid disease. Two hundred fifteen (63%) have normal weight whereas about 53 (15.5%) respondents self-rated their health as very good. Poor self-efficacy to manage hypertension accounts 70% of respondents (Table 1).

**Table 1:** Characteristic of hypertensive patients at Jimma University Specialized Hospital (n = 341)

Variables	Frequency (%)	Variables	Frequency (%)
*Age*		*Ethnicity*	
19–39 years	42 (12.3)	Oromo	200 (58.7)
40–64 years	222 (65.1)	Amhara	51 (15)
65–89 years	77 (22.6)	Tigre	18 (5.3)
*Gender*		Guragie	26 (7.6)
Male	177 (51.9)	Dawuro	17 (5)
female	164 (48.1)	Kafa	17 (5)
*Education*		Yem	4 (1.2)
		Tanbaro	3 (0.9)
		Sulte	5 (1.5)
Illiterate	149 (43.7)	*Average monthly income (ETB)*	
Read and write	35 (10.3)	< 500	61 (17.9)
Primary	80 (23.5)	501–1500	122 (35.8)
Secondary	43 (12.6)	1501–2500	82 (24)
College/above	34 (10)	2501–3500	38 (11.1)
*Religion*		> 3501	38 (11.1)
Muslim	186 (54.5)	*Live alone*	
Orthodox	99 (29)	Yes	24 (7)
Protestant	55 (16.1)	No	317 (93)
Wakefata	1 (0.3)	*Social support*	
*Occupation*		Low	114 (33.4)
House wife	82 (24)	Medium	177 (51.9)
Farmer	122 (35.8)	Greater	50 (14.7)
merchant	38 (11.1)	*Marital status*	
Employed	47 (13.8)	Married	279 (81.8)
Retired	32 (9.4)	Single	2 (.6)
Daily laborer	7 (2)	Widow	46 (13.5)
House servant	9 (2.6)		
Students	4 (1.2)		
*Place of residence*		Divorced	14 (4.1)
Rural	159 (46.6)	*BMI*	
Urban	182 (53.4)	16.3–18.499	22 (6.5)
*Time since diagnosis of hypertension*		18.5–24.99	215 (63)
< 3 years	104 (30.5)	25–29.9	92 (27)
≥ 3 years	237 (69.5)	≥ 30	12 (3.5)
*self-reported Comorbidities*		*Self-rated health*	
Diabetes	55 (16.1)	Very good	53 (15.5)
Heart failure	20 (5.9)	Good	141 (41.3)
Kidney disease	26 (7.6)	Fair	113 (33.1)
Liver disease	2 (0.6)	Poor or very poor	34 (10)
Asthma	10 (2.9)	*Self-efficacy*	
Retinopathy	5 (1.5)	Good	103 (30.2)
Neuropathy	3 (0.9)	Poor	238 (69.8)

### Prevalence of self-care practices of hypertensive patients

Of the study participants; 61.9%, 30.5%, 44.9%, 93.5%, 88.3% and 56.9% were reported adherent to medication usage, low salt diet, physical activity, non-smoking, non-alcohol drinking and weight management practices respectively (Table 2).

**Table 2:** Self-care practices of hypertensive patients at Jimma University specialized hospital (n = 341)

Prevalence of self-care practices	
Variables	Frequency (%)
*Medication usage*	
Adherent	211 (61.9)
Non-adherent	130 (38.1)
*Physical activity*	
Adherent	153 (44.9)
Non-adherent	188 (55.1)
*Weight management*	
Adherent	194 (56.9)
Non-adherent	147 (43.1)
*Low salt diet*	
Adherent	104 (30.5)
Non-adherent	237 (69.5)
*Non Smoking*	
Adherent	319 (93.5)
Non-adherent	22 (6.5)
*Moderate alcohol usage*	
Adherent	301 (88.3)
Non-adherent	40 (11.7)

### Predictors of self-care practices

In bivariate logistic regression variables like younger age, female sex, normal weight, hypertension knowledge, self-efficacy, education, time since hypertension diagnosis and marital status were significantly associated with SCPs.

In multivariate logistic regression, normal weight patients were 1.82 times more likely to adhere medication usage practice than over weight respondents (AOR = 1.822, 95% CI 1.073–3.093). However, participants of poor self-efficacy (AOR = 0.407, 95% CI 0.227–0.730) were less likely to adhere medication usage than participants of good self-efficacy.

Participants who get greater social support were 2.81 times (AOR = 2.811, 95% CI 1.209–6.534) more likely adherent to low salt diet than their counterparts.

Female were 3.63 time more likely to non-smoking than male (AOR = 3.626, 95% CI 1.211–10.851).

Respondents having adequate knowledge of hypertension were 2.58 times more likely (AOR = 2.585, 95% CI 1.125–5.940) to adhere practicing physical activity. However, female (AOR = 0.517, 95% CI 0.301–0.887) respondents were less likely to adhere physical activity than male.

Normal weight respondents were 2.22 times more likely (AOR = 2.219, 95% CI 1.218–4.043) to practice weight management. Besides, having good self-efficacy were 2.60 times more likely (AOR = 2.584, 95% CI 1.411–4.731) to maintain their weight than poor self-efficacy (Table 3).

**Table 3:** Predictors of Self-care practices among hypertensive patients at Jimma University specialized Hospital

Variables	Medication usage		Univariate analysis		Multivariable analysis	
	Adherent	Non-adherent	P-value	COR (95% CI)	P-value	AOR (95% CI)
*Age in years*						
19–39 years	32	10	0.022	2.667 (1.151–6.176)	0.064	2.455 (0.951–6.339)
40–64 years	137	85	0.270	1.343 (0.795–2.268)	0.380	1.300 (0.723–2.337)
≥ 65 years	42	35	1.0	1.0	1.0	1.0
*Time of HTN diagnosis*						
< 3 years	74	30	0.020	1.800 (1.096–2.958)	0.092	1.605 (0.926–2.782)
≥ 3 years	137	100	1.0	1.0	1.0	1.0
*BMI*						
16–18.49	14	8	0.360	1.559 (0.603–4.032)	0.098	2.396 (0.851–6.747)
18.5–24.9	142	73	0.024	1.733 (1.075–2.793)	0.026	1.822 (1.073–3.093)
≥ 25	55	49	1.0	1.0	1.0	1.0
*Self*-*efficacy*						
Good	80	23	1.0	1.0	1.0	1.0
Poor	131	107	0.000	0.352 (0.207–0.598)	0.003	0.407 (0.227–0.730)

Variables	Low salt diet		Univariate analysis		Multivariable analysis	
	Adherent	Non–adherent	P–value	COR (95% CI)	P–value	AOR (95% CI)
*Time of HTN diagnosis*						
< 3 years	40	64	0.035	1.689 (1.037–2.753)	0.050	1.752 (0.999–3.074)
≥ 3 years	64	173	1.0	1.0	1.0	1.0
*Social support*						
Low	23	91	1.0	1.0	1.0	1.0
Medium	58	119	0.020	1.928 (1.107–3.358)	0.053	1.837 (0.992–3.401)
Greater	23	27	0.001	3.370 (1.640–6.925)	0.016	2.811 (1.209–6.534)
*HTN knowledge*						
Poor	28	94	1.0	1.0	1.0	1.0
Average	55	127	0.164	1.454 (0.858–2.464)	0.313	1.345 (0.756–2.391)
Adequate	21	16	0.000	4.406 (2.029–9.567)	0.003	3.789 (1.575–9.114)
*Self*-*efficacy*						
Good	49	54	0.001	3.019 (1.849–4.930)	0.001	2.584 (1.477–4.521)
Poor	55	183	1.0	1.0	1.0	1.0

Variables	Physical activity		Univariate analysis		Multivariable analysis	
	Adherent	Non-adherent	P-value	COR (95% CI)	P-value	AOR (95% CI)
*Age in years*						
19–39 years	24	18	0.043	2.207 (1.026–4.745)	0.164	1.864 (0.775–4.480)
40–64 years	100	122	0.261	1.357 (0.797–2.308)	0.345	1.346 (0.726–2.495)
≥ 65 years	29	48	1.0	1.0	1.0	1.0
*Sex*						
Male	93	84	1.0	1.0	1.0	1.0
Female	60	104	0.003	0.521 (0.338–0.804)	0.017	0.517 (0.301–0.887)
*Education*						
Illiterate	47	102	1.0	1.0	1.0	1.0
Read and write	12	23	0.755	1.132 (0.520–2.467)	0.929	0.963 (0.422–2.200)
Primary	43	37	0.001	2.522 (1.442–4.411)	0.077	1.728 (0.942–3.170)
Secondary	29	14	0.000	4.495 (2.176–9.286)	0.002	3.301 (1.529–7.126)
College/above	22	12	0.001	3.979 (1.817–8.711)	0.172	1.912 (0.754–4.846)
*Marital status*						
Married	135	144	1.0	1.0	1.0	1.0
Others	18	44	0.006	0.436 (0.240–0.792)	0.627	0.842 (0.420–1.686)
*HTN knowledge*						
Poor	45	77	1.0	1.0	1.0	1.0
Average	84	98	0.110	1.467 (0.917–2.345)	0.288	1.320 (0.791–2.204)
Adequate	24	13	0.003	3.159 (1.465–6.813)	0.025	2.585 (1.125–5.940)
*Self-efficacy*						
Good	60	43	0.001	2.176 (1.359–3.482)	0.097	1.567 (0.922–2.664)
Poor	93	145	1.0	1.0	1.0	1.0

Variables	Non-smoking		Univariate analysis		Multivariable analysis	
	Adherent	Non-adherent	P-value	COR (95% CI)	P-value	AOR (95% CI)
*Sex*						
Male	160	17	1.0	1.0	1.0	1.0
Female	159	5	0.019	3.376 (1.217–9.379)	0.021	3.626 (1.21–10.851)
*Self*-*rated health*						
Good-very good	190	4	0.001	6.628 (2.193–20.036)	0.012	4.482 (1.39–14.45)
Fair to poor	129	18	1.0	1.0	1.0	1.0
*Social support*						
Low	103	13	1.0	1.0	1.0	1.0
Medium	170	7	0.019	3.126 (1.207–8.093)	0.148	2.246 (0.749–6.732)
Greater	48	2	0.148	3.089 (0.670–14.235)	0.524	1.730 (0.320–9.337)
*Self*-*efficacy*						
Good	102	1	0.026	9.87 (1.310–74.399)	0.052	9.541 (0.98–92.752)
Poor	217	21	1.0	1.0	1.0	1.0

Variables	Non-alcohol usage		Univariate analysis		Multivariable analysis	
	Adherent	Non-adherent	P-value	COR (95% CI)	P-value	AOR (95% CI)
*Education*						
Illiterate	131	18	1.0	1.0	1.0	1.0
Read and write	29	6	0.426	0.664 (0.242–1.819)	0.732	0.817 (0.267–2.250)
Primary	76	4	0.093	2.611 (0.852–7.999)	0.398	1.701 (0.496–5.835)
Secondary	38	5	0.936	1.044 (0.364–2.998)	0.900	1.081 (0.321–3.644)
College/above	27	7	0.198	0.530 (0.202–1.393)	0.036	0.239 (0.063–0.908)
*Presence of DM*						
Yes	43	12	0.014	0.389 (0.184–0.823)	0.282	0.615 (0.254–1.491)
No	258	28	1.0	1.0	1.0	1.0
*BMI*						
16–18.49	19	3	0.675	1.326 (0.354–4.959)	0.581	1.537 (0.334–7.061)
18.5–24.9	196	19	0.029	2.159 (1.080–4.316)	0.084	2.036 (0.909–4.561)
≥ 25	86	18	1.0	1.0	1.0	1.0
*Self*-*rated health*						
Good-very good	178	16	0.024	2.171 (1.107–4.255)	0.198	1.638 (0.773–3.470)
Fair to poor	123	24	1.0	1.0	1.0	1.0

Variables	Weight management		Univariate analysis		Multivariable analysis	
	Adherent	Non-adherent	P-value	COR (95% CI)	P-value	AOR (95% CI)
*Education*						
Illiterate	63	86	1.0	1.0	1.0	1.0
Read and write	21	14	0.061	2.048 (0.967–4.336)	0.095	2.099 (0.879–5.015)
Primary	49	31	0.007	2.158 (1.239–3.758)	0.258	1.467 (0.755–2.849)
Secondary	32	11	0.000	3.971 (1.860–8.476)	0.002	4.146 (1.65–10.405)
College/above	29	5	0.000	7.917 (2.903–21.591)	0.017	4.241 (1.289–13.96)
*BMI*						
16–18.49	13	9	0.273	1.685 (0.663–4.285)	0.058	2.903 (0.964–8.742)
18.5–24.9	133	82	0.008	1.892 (1.178–3.039)	0.009	2.219 (1.218–4.043)
≥ 25	48	56	1.0	1.0	1.0	1.0
*Social support*						
Low	38	76	1.0	1.0	1.0	1.0
Medium	118	59	0.000	4.000 (2.428–6.590)	0.000	4.050 (2.279–7.196)
Greater	38	12	0.000	6.333 (2.971–13.500)	0.000	6.694 (2.733–16.39)
*HTN knowledge*						
Poor	60	62	1.0	1.0	1.0	1.0
Average	106	76	0.120	1.441 (0.909–2.286)	0.303	1.334 (0.771–2.305)
Adequate	28	9	0.006	3.215 (1.401–7.378)	0.011	3.524 (1.331–9.328)
*Self*-*efficacy*						
Good	78	25	0.000	3.215 (1.956–5.504)	0.002	2.584 (1.411–4.731)
Poor	116	122	1.0	1.0	1.0	1.0

## Discussion

Trials showed using SCPs in patients with hypertension have shown reduction in BP, cardiovascular events and total mortality [20].

In this study, the prevalence of SCPs of medication usage was 61.9%, which is similar to studies done in China in which 61.3% of the participants reported taking antihypertensive medications as prescribed [21]. However, this study result is lower than a study done in Tikur Anbessa; Ethiopia in which 69.2% were adherent to medication [22]. This difference might be due to educational variation as some of study participants were illiterate. However, our current result is higher than study done in Nigeria [23]. Normal weight patients adhere to medication use as compared to overweight patients, which is in line with a study done in metropolitan Charlotte area [24].

Importantly in this result, we found the prevalence of SCP of adherence to low salt diet was 30.5%, which is much lower than the study done in China [21]. This might be the daily consumption of salt per person is high in Ethiopia and most countries [25]. Participants who are less than 3 years since diagnosis to have hypertension were found to be independent predictor of low salt diet practice, which is not consistent with research done in china [21]. The possible reason might be patients unable to go through with diet regimen for long period, which is different from the other family members. In addition, participants with greater social support are independent predictor of self-care practice of low salt diet similar to a study done by Hu et al. [26]. Respondents who have adequate knowledge of hypertension adhere to low salt diet and this is in line with a study done in India [27].

In this study, the prevalence of SCP of adherence to physical activity was 44.9%, which is lower than study done in china were 51.9% of participants engage in physical exercise [21]. The main barriers in practicing physical activity were lack of desire and not convinced of the benefits [28]. Zinat Motlagh et al. [29] found 24.5% of hypertensive patients do physical activity, which is lower than our study. However, this study result is in line with a study done in Black Lion, Ethiopia [30]. Respondents who have secondary education practiced physical activity as compared to illiterate since they learnt benefit of physical activity at school. Female patients were less likely to involve in physical activities than males. This is not in line with the study done in China and Iran [21, 29]. In areas like ours, females are culturally made busy at home activities and they are responsible in making foods for their family.

Non-smoking practice was the most widely practiced SCP among hypertensive patients studied, which accounted for 93.5% respondents. This finding was found to be higher compared to a study done in China and India [21, 27]. This might be due to low prevalence of smoking habit in Ethiopia [31] and females were more likely to adhere to non-smoking practice in our study which is in line with a study done in China [21] and different from a study done in Iran [29]. Women are much less likely than men to report using smoking [32].

Non-alcohol use practice was the second most widely practiced SCP, which account for 88.3% of respondents that is higher than study done in china and India [21, 27]. The possible discrepancy may be low alcohol drinking prevalence and difficulty to afford daily expenditure of alcohol. However, this study is lower than study done in Iran because alcoholic drinks are banned in Iran. However, this finding is in line with a study done in Brazil were 88.7% of respondents adherent to non-alcohol drink [33].

More than half of respondents in this study, 56.9% were adherent to SCP of weight management which is higher than study done by Warren-Findlow and Seymour [17]. In addition, this study result is higher than a study done in Iran were 39.2% managed their weight [29]. Having good self-efficacy encouraged practicing weight management similar to a study done by Warren Findlow et al. [24].

## Conclusion

Self-care practices of low salt diet (30.5%), physical activity (44.9%), medication usage (61.9%) and weight management (56.9%) were low whereas self-care practices of non-alcohol use and non-smoking were good. Self-efficacy was independent predictor of SCPs of low salt diet and weight management. Females were independent predictor of non-smoking.

## Limitation

Recall bias may influence the result this study because data was gathered through a self-report questionnaire. It was difficult to assess the amount of salt intake of the patients.

## Abbreviations

AOR: adjusted odds ratio
BP: blood pressure
DASH: dietary approaches to stop hypertension
ETB: Ethiopian Birr
H-SCALE: hypertension self-care activity level effects
JUSH: Jimma University Specialized Hospital
SCP (s): self-care practice (s)
